# Interplay of Good Bacteria and Central Nervous System: Cognitive Aspects and Mechanistic Considerations

**DOI:** 10.3389/fnins.2021.613120

**Published:** 2021-02-11

**Authors:** Mahmoud Salami

**Affiliations:** ^1^Physiology Research Center, Institute for Basic Sciences, Kashan University of Medical Sciences, Kashan, Iran; ^2^Department of Neuroscience, Graduate School of Medicine, Kyoto University, Kyoto, Japan

**Keywords:** cognitive brain function, gut microbiota, neurological disorders, probiotics, dysbiosis, eubiosis, learning and memory

## Abstract

The human gastrointestinal tract hosts trillions of microorganisms that is called “gut microbiota.” The gut microbiota is involved in a wide variety of physiological features and functions of the body. Thus, it is not surprising that any damage to the gut microbiota is associated with disorders in different body systems. Probiotics, defined as living microorganisms with health benefits for the host, can support or restore the composition of the gut microbiota. Numerous investigations have proved a relationship between the gut microbiota with normal brain function as well as many brain diseases, in which cognitive dysfunction is a common clinical problem. On the other hand, increasing evidence suggests that the existence of a healthy gut microbiota is crucial for normal cognitive processing. In this regard, interplay of the gut microbiota and cognition has been under focus of recent researches. In the present paper, I review findings of the studies considering beneficial effects of either gut microbiota or probiotic bacteria on the brain cognitive function in the healthy and disease statuses.

## Introduction

It has long been known that consuming fermented food, especially milk and its products, is beneficial for body health (Guo et al., 2014; McFarland, 2015). The importance of intestinal microbes to human health was first perceived by Élie Metchnikoff early in the twentieth century, who attributed unusually long lives to using the dairy products consisting microorganisms (Podolsky, 2012). Despite public attention about the importance of the exogenous microorganisms, however, it was later cleared that an extreme number of microorganisms, mostly different strains of bacteria, coexists in the gastrointestinal tract. This community of microorganisms is named “gut microbiota.” During several decades and particularly recent years, it appeared that we cannot only ignore our intestinal guests but also we intensively need these commensal populations for a normal live (Al-Asmakh and Hedin, 2015; McKenney and Pamer, 2015). Actually, growing documents indicate that without regulatory effects of the gut microbiota on different systems, our body would be a target of many diseases (Ding et al., 2017; Zhuang et al., 2018; Lubomski et al., 2019; Painold and Morkl, 2019). Hence, it is not surprising that probiotics, as helpful microorganisms, was considered to be used to restore damaged gut microbiota or further support it (Kwok et al., 2014). Although in primary studies the effectiveness of the gut microbes was mostly considered in the gastrointestinal tract and the digestive diseases emerged from impaired gut microbiota (e.g. irritable bowel syndrome), however, it was later found that, through production of a variety of bioactive substances, the gut microbiota considerably impact different body organs. Especially, the intestinal bacteria has a mutual relation with the nervous system so that the current findings imply that an intact gut microbiota is required for proper brain function (Grenham et al., 2011). In this context, the gastrointestinal tract is known to be the origin of some neurological disorders (Hu et al., 2016). Necessity of coexistence with the gut microbiota and the helpful effects of probiotic supplements on the nervous system are being under intensive research. Considering animal models as well as some human brain disorders, broad range of brain cognitive functions has been subject of experimental and clinical investigations. Focusing on the animal and human studies, this review answers how the gut microbiota as well as the probiotic bacteria influences cognitive functions. Particularly, the different mechanisms through which the beneficial bacteria impact the cognitive phenomena are discussed. The effect of bacteria on other brain actions and related mechanisms are not considered.

## Gut Microbiota

Contents of bowel have long been considered simply waste products, ignoring a vital community whose close interactions with body affect our life in various levels (Tillisch, 2014). Even after finding beneficial effect of intestinal bacteria, for a long time, it seemed unlikely that these microorganisms could affect body organs other than the digestive system. However, it is now known that the composition of the gut microbiota affects a wide range of physiological processes (Novotny et al., 2019). Actually, about 90% of human body cells are not human eukaryote cells but of prokaryotic origin (Luckey, 1972; Frank and Pace, 2008; Forsythe and Kunze, 2013). The gut microbiota comprises approximately 10^13^ microorganisms (Backhed et al., 2005; Gill et al., 2006). It encompasses almost 1–2 kg in adult humans (Forsythe and Kunze, 2013) that equals weight (1.5 kg) of a normal adult brain (Parent and Carpenter, 1996). From more than 100 bacterial phyla, only a few divisions have been identified in the human gut that includes *Firmicutes*, *Bacteroidetes*, *Proteobacteria*, *Verrucomicrobiota*, *Fusobacteria*, *Cyanobacteria*, *Actinbacteria*, and *Spirochetes* (Backhed et al., 2005); of them, the first and second phyla represent 70% of the total microbiota (Mariat et al., 2009). Colon contains more than 70% of the microorganisms colonizing the gastrointestinal tract (Hold et al., 2002). Additionally, the microbial composition varies between the various parts of the gut (Frank et al., 2007). Moreover, a significant difference exists between the kind of microbiota in the lumen and the microbiota embedded in the mucus layer of the gut (Swidsinski et al., 2005).

### Gut Microbiome

While the human genome hosts 26,600 genes, the gut microbiota encode about 4,000,000 genes (The Human Microbiome Project and Consortium, 2012; Hu et al., 2016). Thus, the total genome of the gut microbiota, what is called “gut microbiome,” exceeds by about 150 times of the number of genes in the human genome. Of these, approximately 55% of genes of the microbiome constitute core metagenome (genes shared among at least 50% of individuals) but the other 45% appear to be unique and/or present in less than 20% of individuals (Petschow et al., 2013). It is surprising that the genome of the intestinal bacteria, which is different than that of humans, encode highly specific enzymes with capability of the intestinal digestion and fermentation (El Kaoutari et al., 2013; Sonnenburg and Sonnenburg, 2014). This explains the human genome-complexity conundrum and, through a symbiotic relationship with the host (Hooper and Macpherson, 2010), have a vital role in normal physiological function (Hu et al., 2016). For these, the gut microbiota is known as “superorganism” (De Filippo et al., 2019) that settles and has a vital role in human health (Jarchum and Pamer, 2011).

### Microbiota-Gut-Brain Axis

The gut and the brain are bidirectionally connected by several pathways including neural, immune, metabolic, and endocrine pathways (Novotny et al., 2019). On one hand, through these pathways, signals from the brain affect the sensory, motor, and secretory modalities of the gastrointestinal tract and, on the other hand, visceral signals from the gut underlie the brain (Grenham et al., 2011). Therefore, a bottom−up influence of the microbiota on the brain function is alongside with a top−down influence of brain on the composition and variety of the gut microbiota. This bidirectional communication, called the “microbiota-gut-brain axis” (Cryan and O’Mahony, 2011), is increasingly recognized as an integral network for regulation of many physiological systems in the human body (Hwang et al., 2019).

### Postnatal Development of the Gut Microbiota

Early postnatal period of development is important in the formation of a healthy intestinal microbiota (Yang et al., 2016). It is shown that bacteria are found in amniotic fluid, placenta, and meconium of newborns. Microbial colonization of the gastrointestinal tract begins very early after birth and develops toward an adult composition by the age of 3 years (Rodriguez et al., 2015). Environmental factors including mode of delivery (cesarean or vaginal) or microbial contamination (Adlerberth et al., 1991) determine primary composition of the newborn gut microbiota (Grenham et al., 2011; Rautava et al., 2012). During postnatal age, the gut microbiota is well adapted to environmental influences and also is susceptible to undesirable changes due to antibiotic use (Yatsunenko et al., 2012; David et al., 2014) or diet where breastfed infants have a different flora than formula-fed ones (Rodriguez et al., 2015). Correspondingly, early postnatal life is a critical period for continued brain development (Koenig et al., 2011; Borre et al., 2014b) during which the genetic codes and physiological activity of synapses influence neuronal wiring (Goodman and Shatz, 1993). This period is sensitive to external environmental signals such as sensory experience (Salami et al., 2000; Fathollahi and Salami, 2001; Talaei et al., 2016) and internal indicators like the gut microbiota (Diaz Heijtz et al., 2011; Borre et al., 2014a). In this period, synaptogenesis continues following birth, and the synaptic connections achieve the highest density at 2 years of the postnatal age (Judas et al., 2011; Borre et al., 2014b).

### Microbe Specificity

**FIGURE 1:**
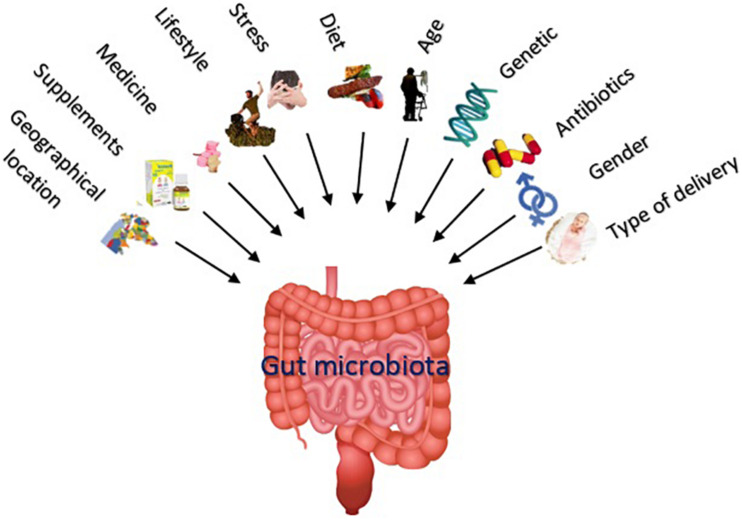
The nature of a steady gut microbiota (including number and variety of the microorganisms) is influenced by various factors including mode of delivery at birth, gender, medications, genetics, age, diet, lifestyle, supplements like prebiotics and probiotics, unwanted impacts like stress, and geographical location.

The nature of a steady gut microbiota of an individual is determined by host, genetics, mode of delivery at birth, lifestyle, environmental factors, diet, eating habits, geographical location, age, gender, microbial transplantation, etc. (de La Serre et al., 2010; Yatsunenko et al., 2012; David et al., 2014; Taneja, 2014). Importantly, the microflora composition is age sensitive, and noticeable differences are evident in microbial profiles during infancy, adolescence, adulthood, and aging. The gut microbiota composition is also influenced by some unwanted impacts such as infection, stress (Yatsunenko et al., 2012; David et al., 2014), and medication use (Koenig et al., 2011). Accordingly, whereas about one-third of microorganism composition of the gut microbiota is common in most people, the rest of two-thirds is specific to each individual (Parashar and Udayabanu, 2017), what is supposed as a “microbial fingerprint” (Franzosa et al., 2015). Figure 1 summarizes the different factors affecting the gut microbiota composition and diversity.

### Eubiosis and Dysbiosis

Many attempts have been devoted to link the gut microbiota with the brain function (De-Paula et al., 2018) as well as the brain diseases (Jiang et al., 2017). Mounting evidence demonstrates that normal function of the brain highly depends on the normal composition of the microbiota, called “eubiosis.” Eubiosis is the status characterized by predominance of potentially beneficial species (“good bacteria,” belonging mainly to bacterial phyla *Firmicutes* and *Bacteroides*) over very low percentage of potentially pathogenic species (“bad bacteria,” belonging to the phyla *Proteobacteria*) (Zhang et al., 2015). On the other hand, decreased intestinal biodiversity or increased pathogenic bacteria named “dysbiosis,” leads to some damages to the brain function.

### The Microbiota Modulation

The idea that the intestinal bacteria play considerable roles in the brain function is strongly proposed by the fact that the changes in both abundancy and variety of specific strains of bacteria (Kang et al., 2013; Scheperjans et al., 2015; Tomova et al., 2015) influence the pathophysiology of neurological disorders (Gonzalez-Gonzalez et al., 2018; Gonzalez-Arancibia et al., 2019). Examples are the dysbiosis reported in Alzheimer’s disease (AD) (Zhuang et al., 2018), multiple sclerosis (MS) (Calvo-Barreiro et al., 2018), autism (Finegold et al., 2002; Rosenfeld, 2015; Ding et al., 2017), and Parkinson’s disease (PD) (Scheperjans et al., 2015; Pietrucci et al., 2019). The main factors to initiate the cognitive deficits are depression, specific personality traits, cardiovascular diseases, cardiac dysfunction, insulin resistance, dyslipidemia, sarcopenia, malnutrition, chronic inflammation, and endocrine perturbations such as hypogonadism and hypovitaminosis D. The majority of these factors are related to changes in composition of the gut flora (Panza et al., 2019).

**FIGURE 2:**
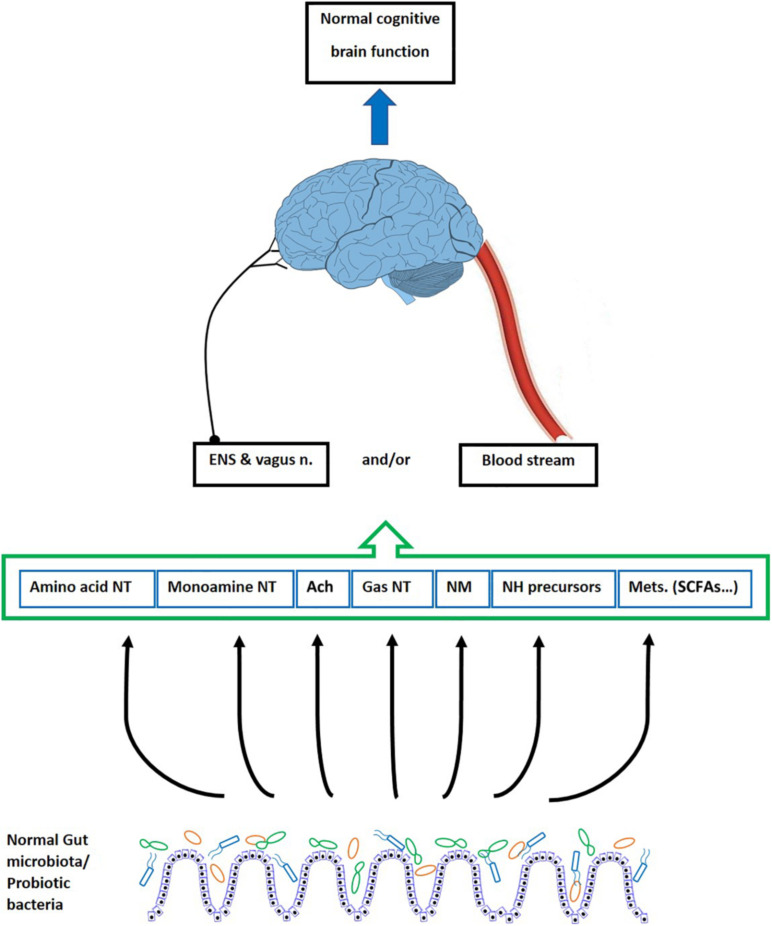
The effect of gut microbiota on the cognitive brain function. The gut microbiota and probiotic bacteria produce neurotransmitters, some neurotransmitter precursors, neuromodulators, and various metabolites. These bioactive factors underlie the cognitive processes of the brain including learning and memory. They reach the brain through blood vessels, cross the BBB, and directly affect the brain function. However, since the BBB is impermeable to polar molecules, it is suggested that some productions of the bacteria impact the brain action indirectly via modulating the ENS and the vagus nerve. ENS, enteric nervous system; n, nerve; NT, neurotransmitter; Ach, acetylcholine; NM, neuromodulators; NH, neurohormones; Mets, metabolites; SCFAs, short chain fatty acids.

Various factors indicate relevancy of gut microbial alterations with behavioral deficits. They include enhanced intestinal permeability and increased inflammation (Krabbe et al., 2005; McCusker and Kelley, 2013; Takechi et al., 2013; Bruce-Keller et al., 2016), impaired synaptic plasticity (Bercik et al., 2011a; Diaz Heijtz et al., 2011), and alterations in neurotransmitters, receptors, and metabolites (Neufeld et al., 2011). In this context, the changes occurring in the brain following the gut microbiota manipulation mostly comes from humoral, hormonal, or neuronal routes. This occurs through signals (i.e., nerves, second messengers) within the gut, conducted directly to the brain, or by bioactive molecules delivered to the blood stream and traveling up to the brain (Figure 2) (Rhee et al., 2009; Dinan et al., 2013). In addition, several studies have demonstrated that changes in the gut microbiota alter the expression of various genes and adjust the neurotransmitters and synaptic associated proteins that, in turn, impact the brain development and function (Diaz Heijtz et al., 2011; Distrutti et al., 2014). Accordingly, it is proved that the gut microbiota alteration can influence memory (Gareau et al., 2011), exploratory behavior (Bercik et al., 2011a), anxiety, and depression (Bercik et al., 2011b; Bravo et al., 2011).

Different approaches have been used by researchers to manipulate intestinal fora to disclose connections between the gut microbiota and normal functions of the brain (such as learning and memory, emotion, and cognition) or pathological conditions (such as brain developmental disorders, mood disorders, and anxiety) (Hooks et al., 2018). Generally, these methods, either negative or positive interventions, include engaging germ-free animals, infections (Gareau et al., 2011; Gareau, 2014), antibiotic administration, dietary manipulations (Li et al., 2009; Ohland et al., 2013; Desbonnet et al., 2015; Frohlich et al., 2016), treating with probiotics (Davari et al., 2013; Ohland et al., 2013), prebiotics, synbiotic (Romo-Araiza et al., 2018), and fecal microbiota transplantation (Parashar and Udayabanu, 2017). Furthermore, aging (Claesson et al., 2011; Leung and Thuret, 2015; Zapata and Quagliarello, 2015; Arboleya et al., 2016), obesity (Ley et al., 2006; Turnbaugh et al., 2006; Turnbaugh and Gordon, 2009), and stress (Gareau et al., 2007; O’Mahony et al., 2009) are known to change the microbiota and have a strong association with dysbiosis of the gut microbiome.

Methodologically, germ-free animals and antibiotic-induced dysbiosis are known as the main approaches by which causality in microbiota-gut-brain axis is established. Therefore, here I consider evidence on the two animal models of research being used for assessment of the intestinal microbiota.

### Germ-Free Animals

One way to address how the intestinal microbes underlie the central nervous system (CNS) is to examine the events when the microbiota is absent. Germ-free animals are animals that are born, bred, and raised free of all microorganisms. They can be used as an experimental tool for evaluating the significance of microbial colonization (Gonzalez-Arancibia et al., 2019). Studies on germ-free animals have shown abnormal brain development, as signed by the dysfunction in neuronal plasticity (Ogbonnaya et al., 2015), neuroprotection (El Aidy et al., 2015), neurotransmission (El Aidy et al., 2015), myelination (Hoban et al., 2016), neurotrophin expression (Gareau et al., 2011), stress hormone signaling (Sudo et al., 2004), and various behavioral abnormalities (Luczynski et al., 2016). These considerations remark that the gut microbiota is essential for the normal cognitive development (Gareau et al., 2011) and, abnormal behavioral development will be occurred in lack of, or any damage to, the microbiome.

In the molecular level, evidence from the germ-free animals indicate changes in gene expression of brain-derived neurotrophic factor (BDNF) (Bercik et al., 2011a; Desbonnet et al., 2015; Kiraly et al., 2016), vasopressin, oxytocin, serotonin transporter (Desbonnet et al., 2015), tyrosine kinase receptor B (TrkB), α-amino-3-hydroxy-5-methyl-4-isoxazolepropionic acid (AMPA) receptor (Kiraly et al., 2016; Gonzalez-Arancibia et al., 2019), 5HT1A serotonin receptor, and *N*-methyl-D-aspartate (NMDA) receptor subunit NR2B mRNA (Neufeld et al., 2011).

The gut microbiota produces bioactive substances (see below) which are shown to be altered in the germ-free animals. The germ-free mice have shown higher turnover rates of dopamine, norepinephrine, and serotonin in the striatum (Diaz Heijtz et al., 2011; Nishino et al., 2013), the brainstem, and the medial prefrontal cortex (Nishino et al., 2013). On the other hand, the germ-free animals exhibit a decreased dopamine turnover rate in the striatum, the hippocampus, and the frontal cortex in comparison with the controls (Crumeyrolle-Arias et al., 2014). Changes in the serotonergic system are shown in the germ-free animals where they display increased precursor and metabolite of serotonin (Clarke et al., 2013) and increased expression of BDNF (Bercik et al., 2011a). The development of hypothalamus-pituitary-adrenal (HPA) axis in the germ-free mice is shown to be abnormal, leading to altered response to stress (Sudo et al., 2004). Based on these, it is concluded that behavioral deficits including cognitive impairments (Gareau et al., 2011), observed in the germ-free animals, demonstrate a potential role for the beneficial microbes in regulating the memory and cognition (Gareau, 2016). Thus, regulating the gut microbiota may be a promising strategy for treatment of cognitive deficits.

### Antibiotics-Treated Animals

The gut microbiota composition is significantly modified by antibiotics, and they are used increasingly as a dysbiosis model. Antibiotic administration from postnatal age forward considerably reduces total bacterial counts and decrease microbiota diversity. For instance, Desbonnet et al. (2015) showed that antibiotic administration led to dysbiosis which was associated with cognitive deficits and decreased expression of hippocampal BDNF. Frohlich et al. (2016) reported that antibiotic-induced dysbiosis impaired novel object recognition memory of mice. This cognitive impairment was related to reduced bacteria-derived metabolites of colon, altered lipid species, and converted microbe-derived molecules in plasma, changing expression of cognitive signaling molecules such as BDNF, NMDA receptor subunit 2B, and tight junction protein expression (Frohlich et al., 2016). Wang et al. (2015) reported that administration of ampicillin in rats resulted in disrupted gut microbiota, impaired spatial memory, and increased anxiety-like behavior.

### The Gut Microbiota Function

The gut microbiota has both positive and negative effects on the human body (Turnbaugh et al., 2006). Through producing a wide range of bioactive substances, the gut microbiota can significantly modulate the human behavior, physiology, and biology (Parashar and Udayabanu, 2017). Current evidence demonstrates that the composition and quantity of the gut bacteria can affect cognitive brain function. Based on this, numerous studies indicate that intestinal homeostasis has a direct influence on the brain function (Cryan and Dinan, 2012; Borre et al., 2014a; Dinan et al., 2014; Carabotti et al., 2015). Particularly, whereas cognition was first thought to be solely regulated by the CNS, it is now known that some other players, including the intestinal microbiota, take a role in cognition (Gareau, 2016). The beneficial effect of either natural gut microbiota or supplementary-administered probiotic bacteria, on the cognitive brain function will be later discussed.

## Probiotics

### History of Probiotic Use

“Probiotics,” which was initially established by Lilly and Stillwell in 1965, are defined as live microorganisms with the capability of promoting health to human and animal hosts when administered in adequate amount (Sherman et al., 2009). They make a dexterous interaction with the microorganisms naturally existing in the gastrointestinal tract. Due to increased awareness about the health promoting effect of probiotics, ingestion of these useful bacteria is rapidly increasing, which is widely reflected in scientific literature. In addition to the foods that are traditionally fermented with probiotics such as yogurt and some other dairy products, new forms of probiotic products are emerging including probiotic capsules and pills, fruit juices, biscuits, breads, cereals, sausages, cookies, candy, sweets, etc. It is estimated that global probiotics markets have earned 35 billion dollars in 2015 and predicts that it will reach 66 billion dollars by 2024 (Jabr, 2017). The strains of lactic acid bacteria including *Lactobacilli* and *Bifidobacteria* are commonly used probiotics which are also normally found in healthy gut (Bodera and Chcialowski, 2009; Azad et al., 2018).

The genus *Lactobacillus* includes various gram-positive bacteria. They can convert hexose sugars to lactic acid, thus producing an acid environment which prevents the growth of several species of harmful bacteria (Makarova et al., 2006). In humans, the *Lactobacilli* are particularly present in the gastrointestinal tract and vagina (Walter, 2008) and, along with the *Bifidobacteria*, are the first bacteria which are colonized in the gut at postnatal age (Walker, 2013). The Lactobacilli such as *Lactobacillus acidophilus*, *Lactobacillus casei*, *Lactobacillus paracasei*, *Lactobacillus rhamnosus*, *Lactobacillus delbrueckii* subsp. *bulgaricus*, *Lactobacillus brevis*, *Lactobacillus johnsonii*, *Lactobacillus plantarum*, and *Lactobacillus fermentum* are frequently used as probiotics.

The genus *Bifidobacterium* consists of various gram-positive anaerobic bacteria which also inhabit the gastrointestinal tract (Chen et al., 2007). The *Bifidobacteria* such as *Bifidobacterium longum*, *Bifidobacterium bifidum, Bifidobacterium adolescentis*, *Bifidobacterium infantis*, *Bifidobacterium animalis*, *Bifidobacterium lactis*, and *Bifidobacterium breve* are considered important probiotics.

These two main genera have various culture conditions. The probiotics efficiently interact with the gut microbes and offer host’s health benefits (Rinaldi et al., 2003). In recent years, a growing body of studies has been devoted to reveal if favoring the gut microbiota by probiotic bacteria impacts the brain functions. Accordingly, probiotics have been found to influence dysfunction of the CNS in neurological disorders by increasing both diversity and count of the intestinal bacteria population (Kwok et al., 2014).

### Psychobiotics

Dinan et al. established the term “psychobiotics” explaining the probiotics that have potential application in treating the psychiatric disorders (Dinan et al., 2013). Through different signaling pathways, the psychobiotics play an important role in controlling the neural excitatory inhibitory balance, mood, cognitive functions and, learning and memory processes (Heldt et al., 2007; Lu et al., 2008; Martinowich and Lu, 2008).

### Probiotic Functions

It is proposed that functions of probiotics can be classified as trophic, protective and metabolic ones (Kusku-Kiraz et al., 2018). Through fermenting “prebiotics,” which are known as non-digestible foods, probiotics appear to have numerous functions including anti-inflammatory, antidiabetic, antiobesity, antipathogenicity, angiogenic, anticancer activities, and neuroprotective properties (George Kerry et al., 2018). Within the digestive system, through adhering to intestine, probiotics stimulate, modulate, and regulate various functions including digestion, competitive exclusion of pathogens, epithelial innate immunity, metabolism, and gut-brain communication (Kristensen et al., 2016; Rao et al., 2016). The probiotic bacteria are reported to produce many non-viable metabolic byproducts such as neurotransmitter, neuromodulators, antioxidants, acetaldehydes, diacetyl, ethanol, organic acids, and hydrogen peroxide (discussed later). These substances have been found to be non-toxic and non-pathogenic, as well as resistant to enzyme systems in mammals. Particularly, because of their biological activity and inhibitory characteristics against pathogenic bacteria in the host, the probiotics are considered an alternative to antibiotics (Ooi et al., 2015; Islam, 2016).

### Studies on Probiotic Interventions

Assessment of the cognitive functions modulated by probiotics has been the target of vast preclinical and clinical investigations, and research in this field is robustly growing. Experimental tasks measured in the animal studies evaluating probiotic interventions have been spatial memory mainly examined by the Barnes maze and Morris water maze tests and non-spatial memory measured with the T-maze, passive avoidance, novel object recognition task, and fear conditioning tests. Albeit scant, however, attempts have also been dedicated to discover the cognitive functions of the probiotics in the human being.

The investigations measuring the cognitive indices have been carried out in both healthy and diseases conditions. The cognitive aspects of probiotic actions mostly include those performed in animal models of brain diseases and human neurological disorders which are directly relevant to cognition (e.g., AD and dementia). However, cognitive measurements are also considered in some other brain (such as epilepsy, stress, MS, and PD) as well as non-brain diseases such as irritable bowel syndrome (IBS) and diabetes mellitus.

In probiotic interventions, using the monospecies bacteria helps to contribute the observed effects to a specific bacterium. However, studies have shown that the multispecies probiotics (consisting of a combination of various strains of specific genera) can increase effectiveness. It can be due to an additive effect of specific strain characteristics like colonization of different niches, induction of an optimal pH range, and enhanced adhesion, in comparison with monospecies supplements (Timmerman et al., 2004; Chapman et al., 2011).

The dose of probiotic bacteria used in animal researches ranges from 10^7^ to 10^11^ colony forming units (CFU), mostly applying 10^9^ or 10^10^ CFU. The duration of the probiotic administration in different studies vary from 1 to 11 weeks. In the human studies, the concentration of probiotic supplementation has been between 10^7^ and 3.63 × 10^10^ CFU, mostly applying 10^9^ and 10^10^ CFU. The treatments have lasted from 3 to 8 weeks (Wang et al., 2016).

### Positive Effect of Probiotics on Cognition

#### Healthy Subjects

Numerous studies have shown that probiotic administration favorably affect cognition in control animals. Using the novel object recognition task and the Barnes maze tests, Savignac et al. (2014) found that *Bifidobacterium longum* improve the cognitive function in healthy Balb/c mice. Bravo et al. (2011) reported that *Lactobacillus rhamnosus* increased memory consolidation in the normal healthy animals in the stress-induced hyperthermia, the forced swim test, and the elevated plus maze. *Bifidobacterium longum* 1714 substantially enhanced the learning and memory capabilities evaluated by fear conditioning test, novel object recognition task, and Barnes maze test (Savignac et al., 2015). A mixture of probiotic bacteria enhanced amplitude of the potentiated responses recorded in the CA1 region of hippocampus in the normal reared rats (Rezaeiasl et al., 2019).

The favorable effect of probiotics on the cognitive function in healthy humans is also considered in several studies. Using a multistrain probiotic including different species of *Lactobacilli* and *Bifidobacteria* in a healthy adult population, Steenbergen et al. (2015) demonstrated an improved cognition using Leiden index of depression sensitivity scale. Benton et al. (2007) found that 3 weeks intervention with *Lactobacillus casei* Shirota improved the mood scores in the healthy participants. Messaoudi et al. (2011a) showed that *Lactobacillus helveticus* strain R0052 and *Bifidobacterium longum* strain R0175 improved psychological distress in the healthy volunteers. Moreover, using a questionnaire that measures problem-solving strategies in healthy adult populations, the same formulation of probiotics showed beneficial impact on the overall cognitive function (Messaoudi et al., 2011b). Intake of *Bifidobacterium longum* 1714 by the healthy male volunteers exposed to an acute stress improved the hippocampus-dependent visuospatial memory performance (Allen et al., 2016).

#### Animal Models of Diseases

Different animal models of stress have been subject of probiotic administration. Liang et al. (2015) found that *Lactobacillus helveticus* NS8 improves the object location memory and promotes the object novelty detection in the rats under chronic restraint stress. In a model of hyperammonemia-induced neuroinflammation, rats were administered by the probiotic *Lactobacillus helveticus* strain NS8. They significantly restored cognitive function, reduced the inflammatory markers, and improved anxiety-like behavior (Luo et al., 2014). Gareau et al. (2011) proved that a combination of probiotic bacteria containing *Lactobacillus rhamnosus* R0011 and *Lactobacillus helveticus* R0052 prevented stress-induced impairments in novel object recognition task recognition memory in the animals infected by the intestinal pathogen *Citrobacter rodentium*. Using the novel object recognition task and the Barnes maze, Savignac et al. reported that *Bifidobacterium longum* 1714 and *Bifidobacterium breve* 1205 separately improved learning and memory in the BALB/c mice model of anxiety (Savignac et al., 2015). The treatment of animals with *Lactobacillus rhamnosus* R0011 and *Lactobacillus helveticus* reversed effect of paternal stress on memory and extinction (Callaghan et al., 2016). The administration of probiotics before the induction of colitis restored the colonic inflammation-impaired recognition memory (Emge et al., 2016).

The improving effect of probiotic bacteria on the cognitive behaviors are also assessed in other animal models. Milk fermented with *Lactobacillus helveticus* markedly improved the impaired learning and memory in a mouse model of dementia induced by scopolamine (Ohsawa et al., 2015). Wang et al. (2015) demonstrated that *Lactobacillus fermentum* NS9 administration restored ampicillin-induced impairment in the memory retention. While infection with *Citrobacter rodentium* or chronic treatment with antibiotics in mice decreased both the working and non-spatial memories, the probiotic administration prevented the behavioral changes (Gareau et al., 2011). Using Morris water maze, Musa et al. (2017) found that *Lactobacillus fermentum* or *Lactobacillus casei* attenuated the lipopolysaccharide-induced memory impairment. Beilharz et al. (2018) showed that pre-exposure to a probiotic mixture (VSL#3) could prevent diet-induced memory spatial memory deficits in the rats introduced to the place task. In an animal model of AD, we showed that a cocktail of probiotics restored the impaired learning and memory in the beta amyloid (Aβ)-injected rats (Rezaeiasl et al., 2019). In addition, in a series of experiments, we demonstrated that formulations of probiotics significantly improve the spatial memory examined by the Morris water maze in the ethanol treated (Hadidi Zavareh et al., 2020), and the diabetic (Davari et al., 2013), stressed (Hadizadeh et al., 2019), and epileptic (Bagheri et al., 2019; Tahmasebi et al., 2020) animal models.

The probiotic treatments have also improved cognition in experiments on non-rodent subjects. Parois et al. (2017) reported that the probiotic *Pediococcus acidilactici* improved memory in STI quail, strengthening the idea that the influence of the gut microbiota on the host behavior and memory observed in mammals can also be shared by birds. Lim and Lim (2017) also showed that Zebrafish fed with the two lactic acid bacteria strains *Pediococcus acidilactici* (JN039350) and *Lactobacillus plantarum* (JN039358) display improved spatial learning and memory.

### Effect of Probiotics on Microbiota

As previously pointed out, the natural composition of microbiota, which is established soon after birth, is vital to the normal action of the brain. Hence, it is not surprising that alterations in composition of the intestinal bacteria community contribute to several brain disorders. Growing evidence suggest that, *via* modification of the composition and diversity of microbiome, the probiotics can restore memory and related brain mechanisms when the gut microbiota is robustly dysregulated by different factors (Distrutti et al., 2014; Jeong et al., 2015a). It is reported that, by decreasing the coliform counts and increasing *lactobacilli* and *bifidobacteria* counts, probiotics can prevent Aβ-induced memory deficit (Athari Nik Azm et al., 2018). Gareau et al. (2007) demonstrated that treatment of *Citrobacter rodentium*-infected mice with a combination of probiotic bacteria optimized the *Firmicutes* and *Eubacterium rectale* but increased the *Bacteroides* group. In contrast, there are some evidences against these findings. Suez et al. (2018) reported that the probiotics did not reliably colonize the mice gut and only had a limited colonization in humans, indicating that the helpful effects of probiotics on the brain may not be occurring *via* only changes in the composition of gut microbiome (McNulty et al., 2011; Kristensen et al., 2016). In this context, even some apprehensions have been raised about the potential negative effect of probiotics on microbiota (Slashinski et al., 2012). For explanation of such complications, it is suggested that behavioral and neurological changes can be related to indirect causal routes rather than necessarily direct action of the specific strains of the probiotic bacteria (Sampson and Mazmanian, 2015).

## Clinical Considerations

Compared with the studies on animal models of brain diseases, due to some limitations, much less researches have been carried out on human subjects. However, evidence indicating a link between favorable effect of the gut microbiota and probiotics on cognition is growing up. Here, I review how the gut microbiota or the probiotic bacteria underlie cognitive function in human subjects.

Along with increased average of lifespan, prevalence of brain dysfunction, including neuropsychiatric and neurodegenerative disorders, is expected to be increased (Smith, 2011). Searching a link between the gut microbiota and the neurological disorders indicate that change in diversity and individual genus abundance of the gut microbiota impact the symptoms of the diseases (Kang et al., 2013; Scheperjans et al., 2015; Tomova et al., 2015). Interestingly, although the neurodegenerative diseases display a shared immunological basis, however, different alterations are evident in gut microbiota in the diseases (Forbes et al., 2018). This part of this review focuses on relevancy of some cognition-associated brain diseases, particularly the AD, with the gut microbiota and probiotic bacteria.

### Alzheimer’s Disease

Through alteration of host neurochemistry, the gut microbial metabolites may increase or decrease the risk of AD. Zhuang et al. (2018) found that the gut microbiota composition in the AD patients was different from that in healthy people. One of the pathological features of the AD is the formation of extracellular Aβ plaques in the brain. *Escherichia coli* and *Salmonella enterica* produce amyloid proteins and, hence, can contribute to the pathogenesis of AD (Tse, 2017). Therefore, it is believed that the gastrointestinal tract may be a source of AD, and it is strictly connected to gut microbiota disproportion (Hu et al., 2016). On the other hand, due to their anti-inflammatory and antioxidant properties, probiotics have been considered for their beneficial effects against the onset, manifestations, and concomitant diseases of neurodegenerative disorders. The effect of probiotics on different aspects of the AD has been examined in several animal models. Treatment of D-galactose-induced animal model of AD with *Lactobacillus plantarum* MTCC1325 improved the cognition deficits and restored the acetylcholine concentration and the histopathological features to a normal condition (Nimgampalle and Kuna, 2017). Bonfili et al. (2017) demonstrated that a probiotic supplement (SLAB51) decreased the number and size of Aβ plaques in 3xTg-AD mice in the early stage of AD. The intervention also counteracted the typical morphological alterations of AD, such as reduction in brain weight, the decline of cortical areas, and the general brain damage and shrinkage. Furthermore, *Lactobacillus helveticus* ameliorated metabolism of amyloid precursor protein in cell-based assays, improved memory in mice, and reduced Aβ1–40 serum concentration in mice (Ohsawa et al., 2015), therefore, reducing the risk of Aβ formation. Treatment of an AD model of rat with *Lactobacillus fermentum*, *Lactobacillus acidophilus*, *Bifidobacterium longum*, and *Bifidobacterium lactis* decreased the size of Aβ plaque and improved spatial memory, orientation, and mood (Athari Nik Azm et al., 2018).

Oxidative stress play a key role in occurrence of AD (Rinaldi et al., 2003) that can be modified *via* antioxidant or nutritional supplements (Vina et al., 2004). Probiotic treatment is reported to normalize the oxidant malondialdehyde (MAD) and the antioxidant superoxide dismutase (SOD) in an animal model of AD (Athari Nik Azm et al., 2018). In the same way, by activating Sirtuin-1 (SIRT1)-related pathways, the probiotic treatment of transgenic AD mice led to conserved brain function (Bonfili et al., 2017), a protein deacetylase that, through activating SOD2 and catalase, reduces the reactive oxygen species (ROS) levels in the brain (Cheng et al., 2014).

Kobayashi et al. (2017) reported anti-inflammatory effect of *Bifidobacterium breve* A1 against AD. They showed that, in a mouse model of AD, the bacterium ameliorated the neuronal inflammation and inhibited the cognitive dysfunction. It is known that the hippocampal acetylcholine delays the onset of AD. In a rat model of AD, *Lactobacillus plantarum* MTCC induced the production of acetylcholine and reversed the histopathology features in the animals, giving rise to an improvement in behavior and learning skills (Nimgampalle and Kuna, 2017).

Clinical studies addressing the positive effects of probiotics on the people with AD are negligible. In the first clinical trial, I examined the effect of a probiotic formulation on people suffering from AD. The participants receiving *Bifidobacterium bifidum*, *Lactobacillus fermentum*, *Lactobacillus acidophilus*, and *Lactobacillus casei* for 4 months improved the scores gained from “Mini Mental State Examination” (MMSE) cognitive test (Akbari et al., 2016).

Postmortem evaluations of the brains in AD patients indicate a relationship between the AD with increased histone deacetylase (Graff et al., 2012). Moreover, postmortem assessment of the AD brains reveals a decreased level of gamma amino butyric acid (GABA), highlighting the protective nature of this neurotransmitter against the onset of AD. *Enterococcus faecium* CFR3003 and *Lactobacillus rhamnosus* GG prompt GABA production in different areas of brain including the cortex, hippocampus, and striatum (Divyashri et al., 2015) and, thus, can positively affect the AD.

Based on these considerations, it is proposed that the AD might not consider only as a disease of brain itself, but brain health is closely associated with our whole body. Therefore, understanding the pathogenesis and developing therapies systemically for the AD and also other neurodegenerative diseases is required (Wang et al., 2017; Lionnet et al., 2018).

### Other Neurological Disorders

Some other brain diseases including the neurodegenerative and neurobehavioral disorders are also subjected to altered gut microbiota. Abundant researches have been devoted to examine if the probiotic bacteria can contribute to treatment of neurological diseases; however, only findings on the cognitive function of brain are considered in this review.

Multiple sclerosis is a chronic, demyelinating and immune-mediated inflammatory neurodegenerative disease characterized by damage to the myelin sheaths of axons in the CNS. Through modulating the host’s immune system, altering the integrity and function of the blood–brain barrier (BBB) and triggering autoimmune demyelination, the gut microbiota appears to play an important role in the pathogenesis of the MS (Calvo-Barreiro et al., 2018). Many researchers have attempted to examine if the gut microbiota modulation can relieve the MS symptoms; however, those considering cognitive aspects are very scant. In a series of clinical trials, we found that 12 weeks probiotic supplementation (*Lactobacillus acidophilus*, Lactobacillus *casei*, *Bifidobacterium bifidum*, and *Lactobacillus fermentum*) positively affected some symptoms including mental health, inflammatory factors, and MDA levels in the MS patients (Kouchaki et al., 2017; Tamtaji et al., 2017; Salami et al., 2019). However, in an animal model of MS, Goudarzvand et al. (2016) reported that probiotic administration showed insignificant effect on spatial memory of the animals.

Autism spectrum disorder is a heterogeneous neurodevelopmental disorder with stereotyped behavior, poor communication skill, and social withdrawal disorder. Several studies have documented dysbiosis of the gut microbiota in the autism patients (Finegold et al., 2002; Rosenfeld, 2015; Ding et al., 2017). The disease has also been target of probiotic therapy. A probiotic formulation consisting of *Lactobacillus acidophilus*, *Lactobacillus rhamnosus*, and *Bifidobacteria longum* improved behavior in the autism cases (Shaaban et al., 2018). West et al. (2014) also reported improved behavior in the autism patients after treating with *Lactobacillus acidophilus*, *Lactobacillus casei*, *Lactobacillus delbrueckii*, *Bifidobacteria longum*, and *Bifidobacteria bifidum*. In contrast, Slykerman et al. (2018) found that a probiotic mixture consisting of *Lactobacillus rhamnosus*, *Bifidobacteria animalis*, and *Bifidobacterium lactis* HN019 worsen behavior of the autist participants.

Parkinson’s disease is a chronic and progressive neurodegenerative disorder principally caused by the loss of dopaminergic neurons in the nigrostriatal pathway. This disorder displays an array of motor as well as non-motor symptoms. In majority of cases, non-motor symptoms precede the motor symptoms by years (Postuma et al., 2015). Numerous studies have found significant differences in the overall fecal gut microbiota composition in the PD patients (Lubomski et al., 2019). Some strains of bacteria are upregulated and some are downregulated in the altered gut microbiota of people with PD (Scheperjans et al., 2015; Pietrucci et al., 2019). Recent animal researches demonstrate that *Lacticaseibacillus rhamnosus* HA-114 (Xie and Prasad, 2020) and *Lactobacillus acidophilus*, *Bifidobacterium bifidum*, *Lactobacillus reuteri*, and *Lactobacillus fermentum* (Alipour Nosrani et al., 2020) improved cognitive deficits in rat model of PD. Despite very scarce evidence so far, however, concerning the direct, as well as indirect findings from the effect of probiotics on behavioral performances in human and non-human researches, probiotics might be useful for relieving the cognitive dysfunction in the patients with PD.

An association between the gut microbiota and psychiatric disorders such as schizophrenia (SCZ), bipolar disorder (BD), and depression has been demonstrated. The SCZ is a devastating and debilitating illness characterized by a set of behavioral abnormalities including cognitive dysfunction, delusions, apathy, psychoses, and withdrawal. Experimental and clinical evidence confirm a link between the SCZ and the gut microbiota. In particular, the SCZ is frequently comorbid with the GI disorders that are associated with gut microbial changes (Fadgyas-Stanculete et al., 2014). Shen et al. (2018) found that the abundance of *proteobacteria* was significantly increased in the SCZ patients. In addition, it is demonstrated that transferring the gut microbiota from fecal microbiome of the SCZ patients to the normal or germ-free mice led to the SCZ-relevant behaviors similar to those in the SCZ rodent models (Zheng et al., 2019). One of the eminent mechanisms involving in the pathogenesis of SCZ is immunoinflammatory response (Watanabe et al., 2010). Thus, well-known anti-inflammatory effects of the probiotics are auspicious to alleviate psychiatric disorders of the disease. In this regard, study of Tomasik et al. (2015) proved that probiotic (*Bifidobacterium animalis* subsp. *lactis* strain Bb12 and *Lactobacillus rhamnosus* strain GG) treatment improved the status of some immune-related factors as well as BDNF in SCZ patients.

Bipolar disorder is a chronic mental health condition with debilitating psychiatric disorders which is characterized by extreme changes in mood and intensive alterations in energy level. Evidence indicates an altered microbial profile in the patients with BD (Evans et al., 2017). Painold and Morkl (2019) reported a significantly different microbial composition in the BD patients compared with the healthy people. In studies carried out by Reininghaus et al. (2018), people with BD treated by probiotic formulations showed considerable improvements in attention and psychomotor processing, indicating that the probiotic supplementation might improve cognitive function in the BD individuals. Moreover, Dickerson et al. (2018) treated manic patients with probiotics. The intervention was associated with a substantial advantage in time to all psychiatric rehospitalization.

Depression is one of the most common psychiatric disorders described by anhedonia and depressed mood. Preclinical and clinical studies indicate a connection between the gut microbiota and the occurrence of depression. Human studies have proved altered gut microbiota in the patients with depression (Jiang et al., 2015; Aizawa et al., 2016; Kelly et al., 2016). In a chronic restraint stress model, that resembles depressive symptoms, the abundance of some strains of bacteria was changed in the gut microbiota (Wong et al., 2016). Interestingly, transferring the gut microbiota from the depressed humans to the germ-free mice led to depressive-like behaviors in these animals (Marin et al., 2017). On the other hand, in a mouse model of depression, *Bifidobacterium adolescentis* NK98 and *Lactobacillus reuteri* NK33 isolated from feces of healthy humans suppressed the occurrence and development of anxiety/depression (Lee et al., 2019). Probiotic treatment in animal and human studies promisingly has ameliorated depressive symptoms. *Lactobacillus plantarum* displays antidepressant effects in mice with stress-induced depression (Choi et al., 2019). Administration of the probiotics reduced the depressant behavior in ovariectomized (Sovijit et al., 2019), inflammatory bowel disease (Bercik et al., 2011a; Emge et al., 2016), and obesity (Agusti et al., 2018) models of mice.

Chronic consumption of *Bifidobacterium longum* R0175 and *Lactobacillus helveticus* R0052 had favorable effects on the anxiety and depression-related behaviors in healthy humans (Messaoudi et al., 2011a,b). *Bifidobacterium longum* NCC3001 positively affected the depressive behaviors in the patients with IBS (Meyer and Vassar, 2018). Treatment with the probiotic bacterium *Lactobacillus plantarum* 299v improved cognitive performance and decreased KYN concentration in major depressive patients (Rudzki et al., 2019). On the other hand, contrary to the abovementioned points, Luo et al. (2018) reported that germ-free mice show antianxiety and antidepressant-like behaviors.

**FIGURE 3:**
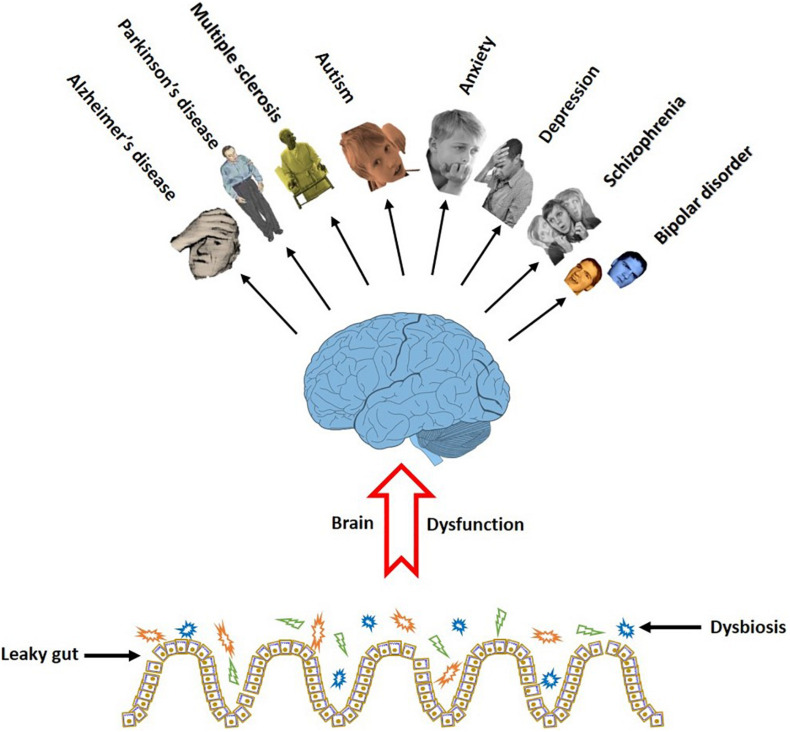
Gut dysbiosis eliminates the favorable effect of the microbiome on the brain function. The cognitive brain dysfunction is a common symptom in a series of neurodegenerative and neurobehavioral disorders in people with damaged gut microbiota.

Taken together, recent findings support a firm link between the neurodegenerative and psychologic disorders with the gut microbiota. Moreover, there are some evidence that probiotic administrations favorably influence the brain diseases. Although most of the neurological disorders are associated with cognitive problems, however, further researches are required to prove the relationship between the gut and probiotic bacteria and the cognitive symptoms. Figure 3 illustrates a link between gut dysbiosis and several neurodegenerative and neuropsychiatric disorders.

### Non-neurological Diseases

Positive effect of the gut microbiota and probiotic bacteria on cognitive brain function is also examined in some non-neurological diseases. IBS is a functional gastrointestinal disease distinguished by chronic abdominal pain and changed bowel habits. Accumulating evidence indicates an abnormal composition or metabolic activity of the gut microbiota in the patients with IBS, demonstrating that the intestinal microbiota plays a pronounced role in IBS (Simren et al., 2013). However, rare studies have considered a relationship between probiotics and IBS-linked mood disorders. Pinto-Sanchez et al. (2017) found that the probiotic *Bifidobacterium longum* NCC3001 reduces depressive scores in the patients with IBS.

It is reported that probiotic supplementation exerts a favorable effect on neurocognitive impairment and neuroinflammation in HIV-1-infected subjects (Ceccarelli et al., 2017). Investigations on obese people showed a rational correlation between gut microbiota composition, cognitive flexibility, attention, and motor speed (Fernandez-Real et al., 2015). Roman et al. (2018) also showed that 8 weeks multispecies probiotic administration improved cognition, specifically decision making and impulsive choice, in patients diagnosed with fibromyalgia. Very recently, the CNS complications associated with the coronavirus disease of 2019 (COVID-19) attributed to either primary CNS involvement or more commonly secondary CNS outcomes are under focus of researches (Najjar et al., 2020).

## Discrepancies

Despite promising results from several studies indicating a favorable effect of the probiotics on, at least, some aspects of the cognitive function, however, some researchers have reported no or even negative effects. Ng et al. (2018) applied a meta-analysis on 10 clinical trials in which people with depressive symptoms were treated by probiotics. There was no significant difference in mood improvement between the treatment and placebo groups (Ng et al., 2018). Consistently, in a clinical trial, Romijn et al. (2017) found no evidence that the probiotic formulation (*Bifidobacterium longum* and *Lactobacillus helveticus*) is useful in the treatment of low mood. In a study by Kelly et al. (2017), *Lactobacillus rhamnosus* (JB-1) failed to modulate cognitive performance in healthy male subjects. Tillmann and Wegener (2019) found that a probiotic mixture consisting of different strains of *lactobacilli* had no effects on cognition in a model of depression. Moreover, Benton et al. (2007) reported that probiotic consumption by people with mild cognitive impairment led to a small decline in performance of an episodic memory task as well as some aspects of long-term memory. In a clinical trial, we found that the probiotic supplementation negligibly affected cognitive scores in very old AD people (Agahi et al., 2018). In a rat model of stress, Goudarzvand et al. (2016) reported that probiotic administration had no positive impact on the performance of spatial memory and learning.

How do these neutral or negative effects of probiotics can be explained? The dissimilarity between outcomes of the different studies could be attributed to the various probiotic mixtures, the disease severity and the duration of the supplement administration. Moreover, in the case of age associated disorders, wrong timing of treatment might be accountable for the failure of supplements where, for instance in rigorous stage of AD, synaptic loss is an irreversible pathological mark (Brewer, 2011). Concurrently, the probiotic treatment at this stage may not be successful in the prevention of the disease process. Besides the abovementioned doubtful evidence on positive efficacy of probiotic in treating brain dysfunction, uncertainty is even more on the effectiveness of probiotics in healthy subjects, and it is believed that the claims that probiotics are beneficial to healthy people are enormously inflated (Jabr, 2017).

Interestingly, most of the findings on ineffective probiotic administration are taken from clinical trials which, in comparison with animal studies, are subjected to more limitations. Indeed, in most, if not all, of human researches, dietary measures are not considered while synergic effect of probiotics with other effective constituents (such as unknown fermented food or substances with symbiotic roles) cannot be ignored (Steenbergen et al., 2015). In addition, precision must be taken in the human cognitive assessments where, answer to questions or filling in forms by the participants can be influenced by culture and level of literacy. Thus, even approved cognitive test (e.g., MMSE as cognitive test in AD patients) must be modified and adapted to the society. Another limitation in the human researches is problem in performing some verifications (e.g., stool sampling), especially in old and patient people when a proof is necessary to judge if or how much the probiotics affected the gut microbiota. Furthermore, how to assure the participants consumed the probiotics according to research protocol might be questionable, especially in the patients who are not in an appropriate level of alertness or self-care situation. Therefore, on one hand, some cautions must be considered in evaluation of the findings in human researches and, on the other hand, despite such neutral or even negative effects, the possibility that probiotics have cognitive values may not to be closed (Sarkar et al., 2016).

## Gut Microbiota, Probiotics, and Aging

Anxiety and memory deficits are two age-induced common symptoms of the brain dysfunction (Hedden and Gabrieli, 2004; Wolitzky-Taylor et al., 2010). Therefore, seeking alternative treatments for memory impairment and age-related anxiety is necessary. The commensal microbiome endures changes along with maturation, especially in the elderly (Claesson et al., 2011; Leung and Thuret, 2015; Zapata and Quagliarello, 2015; Arboleya et al., 2016). The composition of intestinal bacteria in aged people is usually influenced by dietary habit, environment, and health status of people (Claesson et al., 2012). Additionally, medication, nutrients malabsorption, and impaired immunity impact composition of the gut microbiota (Biagi et al., 2013). During aging, the composition of gut microbiota physiologically endures both reduced species richness and increased interindividual variability (Vaiserman et al., 2017). For instance, the number of *Bifidobacteria* in the fecal microbiota decreases in the elderly. The quantity of *Lactobacilli* is found to either increase or bear no differ toward the elderly (An et al., 2018). Interestingly, decreased diversity of the gut microbiota in the old people is usually along with cognitive dysfunctions and reduced brain weight and, a link is proposed between gut microbiota modulation and age-related degenerative cognitive dysfunction (O’Toole and Jeffery, 2015). On the other hand, due to anti-inflammatory activities (Liu et al., 2011), antioxidant activities (Das, 2015), and metabolic regulations (Chen et al., 2018) of the probiotics, they may play a viable role during aging. Consistently, several studies have suggested that probiotics, through elevating the levels of neurotransmitters and neuromodulators (Liu et al., 2016) reduce memory deficits and prevalence of anxiety (Vilela et al., 2017) and, therefore, prevent age-related cognitive declines (Huang and Chen, 2018). Researches focusing on the effectiveness of probiotics in elderly are increasing. Jeong et al. (2015a) demonstrated that a combination of *Lactobacillus plantarum* KY1032 and *Lactobacillus curvatus* HY7601 restored age-reduced spontaneous alternation in the Y maze task in Fischer 344 rats. Furthermore, consumption of *Lactobacillus plantarum* C29 improved the Y maze alternation and acquisition of water maze task in old Fischer 344 rats (Jeong et al., 2015b). A probiotic preparation composed of *Bifidobacterium lactis*, *Lactobacillus casei*, *Bifidobacterium bifidum*, and *Lactobacillus acidophilus* improved the memory deficits, cerebral neuronal and synaptic injuries, glial activation, and microbiota composition in the feces and brains of 9-month-old SAMP8 mice (Yang et al., 2020). The single probiotic *Lactobacillus paracasei* D3-5 improved cognitive functions in old mice (> 79 weeks) (Wang et al., 2020). The middle-aged rats administered by a combination of *Lactobacillus* and *Bifidobacterium* species elicited a slight improvement in spatial accuracy during a new platform location task in a water maze. The animals also showed a pronounced improvement in object novelty detection and improved memory for object-in-place associations (O’Hagan et al., 2017). Ni et al. (2019) demonstrated that, through alteration of the combination and function of the intestinal microbiota, probiotics (BL986 *Lactobacillus casei* LC122 and *Bifidobacterium longum* BL986) display antiaging potentials, which is reflected in improved learning and memory.

Human research also indicates favorable effect of probiotic on cognitive brain function. Kim et al. (2020) reported that administrating a probiotic mixture (*Bifidobacterium bifidum* BGN4 and *Bifidobacterium longum* BORI) promoted mental flexibility and alleviated stress in healthy older (≥ 65 years) subjects. Moreover, *Bifidobacterium breve* A1 (MCC1274) treatment improved memory function in healthy older adults suffering from MCI (Xiao et al., 2020). The probiotic bacterium *Lactobacillus rhamnosus* GG improved cognitive performance in middle-aged and older adults with cognitive impairment (Sanborn and Azcarate-Peril, 2020). Treatment with *Bifidobacterium breve* A1 was efficient in maintaining cognitive function in elderly subjects with memory complaints (Kobayashi et al., 2019). Recent findings hypothesize contribution of the gut microbiota to the course of COVID-19 (Aktas and Aslim, 2020). It is because the diversity of the gut microbiota is decreased in old age and COVID-19 is mainly fatal in elderly patients, hence, the gut microbiota may play a role in this disease (Dhar and Mohanty, 2020).

## Action Mechanism of Bacteria in Cognitive Brain Function

How do beneficial bacteria, whether naturally inhabited in the gut or administered as supplementary support, affect the cognitive function? Numerous mechanisms are proposed by which both intestinal and probiotic bacteria affect the cognitive function. In this way, the beneficial microorganisms engage different divisions of the peripheral nervous system, produce neuroactive substances and their precursors and, numerous metabolites, regulate intestinal mucosa permeability, oxidant/antioxidant balance, and expression of some neurotransmitter receptors, and suppress neuroinflammation and apoptosis.

**FIGURE 4:**
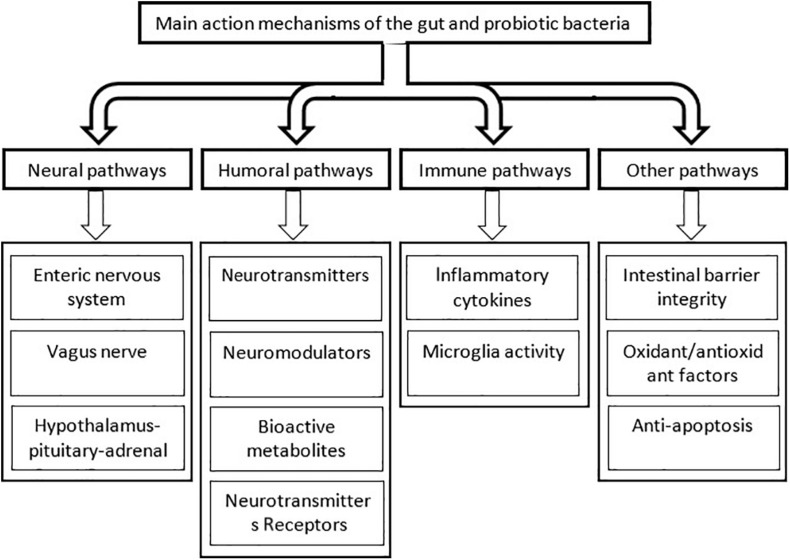
The main pathways by which the intestinal and probiotic bacteria influence the cognitive brain function. For simplicity, interconnections between the different pathways are not shown.

The CNS and different divisions of the autonomic nervous system are suggested to be implicated in communication with the gut microbiota (Cryan and Dinan, 2012). Furthermore, the microbiota produces neuroactive molecules and their precursors which can reach the brain through the afferent autonomic and endocrine pathways (Desbonnet et al., 2008). They can influence cognitive functions through underlying immune activation, receptor activations, and glial cells (Lehnardt et al., 2003; Krabbe et al., 2005; Ait-Belgnaoui et al., 2012; McCusker and Kelley, 2013). Here, the action mechanisms of the helpful bacteria on the cognitive brain function are categorized as the neural, humoral, metabolic, and immune pathways. Figure 4 shows the different mechanisms by which the gut microbiota and probiotic bacteria affect the function of the nervous system.

### Neural Pathways

In the bidirectional network of microbiota-gut-brain axis, the autonomic nervous system, enteric nervous systems, and HPA axis connect the gut to the CNS. This basic anatomical pathway also extends to humoral, endocrine, immune, and metabolic routes (which will be discussed later) of communication allowing the brain to influence the gastrointestinal activities and the gut to influence the brain function including cognition, mood, and mental health. Epidemiological, clinical, and immunological documents prove that the gut microbiota broadly and intensely impact the gut-brain relationship (Appleton, 2018).

#### The Vagus Nerve

The vagus nerve, as a part of the autonomic nervous system, is a major visceral sensory pathway consisting of about 80% afferent fibers. The vagal afferent fibers convey signals from the peripheral organs including the gastrointestinal tract to the CNS. These visceral afferent inputs to the brain can modulate emotion, cognition, and behavior through the brainstem nuclei and ascending cholinergic and noradrenergic projections to the cortex as well (Berntson et al., 2003). In humans, the vagus nerve projects to the medial prefrontal cortex (Mayer et al., 2006) which is associated with the processing of social and affective information (Adolphs, 2001). Experimental findings suggest that the gut-brain communication partly occur *via* the vagus nerve (Ter Horst and Postema, 1997; Tillisch et al., 2013). The study of Bravo et al. (2011) showed that treating mice with a probiotic supplementation beneficially affects anxious and depressive behavior only when the vagus nerve is intact.

#### Hypothalamic-Pituitary-Adrenal Axis

The HPA axis is recognized as the major neuroendocrine system responsible for reaction to the psychological and physical stressors. It includes the master regulator of the HPA axis, corticotropin-releasing hormone (CRH), that induces secretion of adrenocorticotropic hormone (ACTH) that, in turn, stimulates release of glucocorticoids (cortisol in humans and corticosterone in rodents) (Smith and Vale, 2006). It has been shown that, *via* cell survival and initiation of terminal maturation, and remodeling of axons and dendrites, glucocorticoids impact the normal development of the brain (Meyer, 1983). However, both increased and decreased levels of glucocorticoids deteriorate the brain development and function (Lupien et al., 2009).

A part of microbiota-gut-brain communication can take place *via* regulation of the HPA axis. Indeed, for normal cognitive processes like learning and memory, appropriate functioning of the HPA axis is necessary. Evidence indicates that microorganisms can modulate the HPA axis during stress. Based on this, exaggerated HPA responses to stress occur in the stress-exposed germ-free animals (Sudo et al., 2004). Moreover, change in the composition of gut microbiota strongly affects the function of the HPA axis (Hsiao et al., 2013). There are many documents indicating that, *via* influencing the HPA axis, probiotics regulate unusual responses to stress. Ait-Belgnaoui et al. (2012) showed that, through a glucocorticoid-negative feedback regulation, the probiotic bacterium *Lactobacillus farciminis* attenuated the hyper-responsiveness of the HPA axis, and *Bifidobacterium longum* R0175 and *Lactobacillus helveticus* R0052 blocked stress-induced increases in CRH, ACTH, and corticosterone (Ait-Belgnaoui et al., 2014). Even a probiotic formulation consisting of *Lactobacillus helveticus* R0052 and *Lactobacillus rhamnosus* R0011 declined the increased corticosterone levels in stressed newborn rats (Gareau et al., 2007). In an animal model of anxiety, we evaluated pre- and postnatal probiotic treatment on spatial learning and memory of prenatally stress-exposed rats. The intervention favorably affected the spatial learning and memory and normalize the serum concentration of corticosterone (Hadizadeh et al., 2019). Contrary results are also reported where Papalini et al. (2019) found that the useful effects of probiotics on stress-related cognition were not induced by changes in the HPA axis. The regulating effect of probiotics on the HPA axis is also demonstrated in human research. *Lactobacillus plantarum* 299v (Andersson et al., 2016), *Lactobacillus longum* 1714 (Allen et al., 2016), and *Lactobacillus longum* R0175 and *Lactobacillus helveticus* R0052 (Messaoudi et al., 2011a) have been shown to suppress corticosterone levels in healthy volunteers. In addition, administration of *Lactobacillus plantarum* P8 has been shown to be a feasible and natural intervention for alleviation of cognitive symptoms in stressed adults (Lew et al., 2019).

### Neurotransmitters and Neuromodulators

Enteroendocrine cells are dispersed in the intestinal mucosa through which neuroactive molecules are produced in the intestine (Coskun et al., 2013). In addition, beneficial bacteria are able to produce various neurotransmitters, neuromodulators that are involved in the different aspects of brain function. These neuroactive substances which are produced in large quantities can enter the CNS and potentially affect many brain activities including inflammation, stress and anxiety reactions, emotion and mood, and learning and memory (Barrett et al., 2012; O’Mahony et al., 2015; Yano et al., 2015). Different strains of bacteria have been demonstrated to produce neurotransmitters such as GABA, serotonin, catecholamines, melatonin, acetylcholine and histamine (Asano et al., 2012; Barrett et al., 2012; Thomas et al., 2012), and the neuromodulator BDNF.

These bacterially derived transmitters may target the CNS by transport through the circulation; however, it is possible that the neuroactive substances primarily relay signals to the CNS *via* enterochromaffin cells and/or enteric nervous system (Uribe et al., 1994; Stilling et al., 2014). Actually, enterochromaffin cells and enteric cells can release neurotransmitters which themselves, or through second messengers, impact different areas of CNS, *via* somatosensory, spinal, or vagal afferents. Another possibility is that the commensal bacteria may locally in the gut regulate numerous neurotransmitters implicated in cognitive function (Dinan et al., 2013).

#### Serotonin

Serotonin (5-hydroxytryptamine (5-HT)) is a key monoamine in regulating the cognitive function of the brain. Over 95% of the serotonin is produced in the gut, and gut bacteria are important source of serotonin. It has been shown that the blood level of serotonin in the germ-free animals is considerably lower than those with a normal gut microbiota (Yano et al., 2015). The probiotics are shown to influence metabolism of serotonin in the gut and brain tissues. Several bacterial species such as *Lactobacillus lactis* subsp. *cremoris*, *Lactobacillus lactis* subsp. *lactis*, *Lactobacillus plantarum*, and *Streptococcus thermophilus* are shown to synthesize serotonin (Özoǧul, 2004). Moreover, *Bifidobacterium infantis* 35624 produce the serotonin precursor tryptophan (Desbonnet et al., 2008). Li et al. (2019) demonstrated that a combination of two probiotic bacteria *Bifidobacterium longum* and *Lactobacillus rhamnosus* increases serotonin in the frontal cortex and hippocampus.

#### Dopamine

Dopamine has been considered a main regulator of cognitive functions such as memory, attention, decision making, reward, and motivation (Gonzalez-Arancibia et al., 2019). The probiotic bacterium *Bacillus* sp. JPJ produces l-DOPA from l-tyrosine (Surwase and Jadhav, 2011), and different strains of *Enterococcus faecium* convert l-DOPA to dopamine (Villageliu and Lyte, 2018). *Bifidobacterium longum* 1714 is shown to have an impact on dopamine in the mesolimbic pathway, which is also involved in memories related to fear (Zhang et al., 2004; Nestler and Carlezon, 2006; Myers and Davis, 2007). Conversely, in a rat model of depression, *Bifidobacterium longum* R0175 and *Lactobacillus helveticus* R0052 decreased dopamine concentration of plasma (Tillmann et al., 2018).

#### Gamma Amino Butyric Acid

Gamma amino butyric acid is the major inhibitory neurotransmitter in the human CNS. Increase of the GABA in the digestive system correspond increase of the GABA in the CNS. The gut microbiota disturbance that influences the production of GABA in the gut reduces the GABA concentration in the CNS. Strains of *Streptomyces bacillary* (Jeng et al., 2007), *Rhizopus microspores* (Aoki et al., 2003), and *Streptococcus salivarius* (Yang et al., 2008) produce GABA. In addition, different species of the probiotic bacteria such as *Bifidobacterium dentium*, *Lactobacillus brevis*, *Bifidobacterium infantis*, and *Bifidobacterium adolescentis* are able to metabolize glutamate to GABA (Barrett et al., 2012; Mitew et al., 2013; Paula-Lima et al., 2013; Saulnier et al., 2013). The main factors that affect microbial production of GABA are temperature, pH, and fermentation time (Dhakal et al., 2012).

Dysfunction of the GABAergic neurotransmission may implicate in the cognitive impairment (Lanctot et al., 2004). The GABA signaling dysfunctions are associated to depression, anxiety, defects in synaptogenesis, and cognitive impairment such as AD (Aziz et al., 2013; Hornig, 2013). Consistently, postmortem studies on the brain tissues have shown the reduced GABA concentrations in AD patients (Harding et al., 2017) including in frontal, temporal, and parietal cortices (Lanctot et al., 2004; Solas et al., 2015). Based on this, it is suggested that, by stimulating the conversion of glutamate to GABA, probiotics may reduce the onset of AD (Bhattacharjee and Lukiw, 2013).

#### Glutamate

Glutamate is the key excitatory neurotransmitter in the human brain. The glutamatergic transmission plays vital roles in neurodevelopment, dendrites, and axon development, regulating neuronal survival, synaptic plasticity, and learning and memory (Li and Tsien, 2009; Lakhan and Kirchgessner, 2013). It is also involved in the regulation of the microbiota-gut-brain axis. Different strains of *lactobacilli* such as *Lactococcus lactis*, *Lactobacillus plantarum*, and *Lactobacillus paracasei* and are able to synthesize glutamate (Sanchez et al., 2017; Nakayama et al., 2018). It is demonstrated that some strains of the *lactobacilli* isolated from Asian fermented foods are glutamate producers (Zareian et al., 2012).

#### Acetylcholine

The neurotransmitter acetylcholine is the major factor in several cognitive functions including learning and memory. Considerable loss of the cortical cholinergic innervation in the AD patients as well as the memory impairing effects of anticholinergic drugs in the healthy people indicates involvement of the acetylcholine in cognitive processing (Bartus, 2000). Several strains of bacteria especially *Lactobacillus plantarum* are shown to produce acetylcholine (Stephenson and Rowatt, 1947; Savignac et al., 2014; Roshchina, 2016). In addition, through increased expression of choline acetyltransferase by neurons, *Lactobacillus rhamnosus* GG positively underlies the cholinergic transmission (Chandrasekharan et al., 2019). *Lactobacillus plantarum* MTCC1325 decreases acetylcholinesterase in an animal model AD that, in turn, enhances amount of the acetylcholine (Nimgampalle and Kuna, 2017). It is proposed that, *via* affecting acetylcholine, *Bifidobacterium longum* 1714 may improve memory (Hasselmo, 2006).

#### Gaseous Metabolites

Gaseous metabolites of bacteria such as nitric oxide (NO), hydrogen sulfide, and carbon monoxide are involved in the neural control of gut functions (Savidge, 2011; Farrugia and Szurszewski, 2014). While normal fluctuation of endogenous NO levels is needed for physiological functions of neurons including the pathways involving in memory consolidation, aberrant NO implicate in some cognitive disorders, such as AD. Enhanced amount of NO also mediates axonal degeneration, provokes neuroinflammation, and downregulates secretion of the important cognitive neuromodulator BDNF (Tse, 2017).

#### Brain-Derived Neurotrophic Factor

Brain-derived neurotrophic factor, as the most important neurotrophic factor, has effects on neurodevelopment and survival, differentiation, and synaptogenesis. The BDNF helps in the enhancement and maintenance of the hippocampal LTP, as the main candidate mechanism of learning and memory; therefore, it takes a considerable role in the synaptic plasticity as well as the cognitive function (Pang and Lu, 2004). The gut microbiota dysbiosis has been associated with decreased levels of BDNF in the hippocampus and cortex, leading to cognitive disorders (Bercik et al., 2011a). The level of BDNF has been found to be diminished in brain and serum of people with SCZ, AD, and anxiety (Carlino et al., 2013; Lu et al., 2013; Mitew et al., 2013). Aging is also characterized by decreased concentration of BDNF, suggesting that the salvage of the BDNF could significantly underlies the retrieve of cognitive impairment (Pineda-Rodriguez et al., 2017). It has been shown that the memory improving effect of probiotics accompany increased level of BDNF in the hippocampus (O’Sullivan et al., 2011; Corpuz et al., 2018). In line with this, evidence indicates that chronic treatment with *Lactobacillus paracasei* K71 may prevent age-dependent cognitive decline by upregulating the BDNF expression in the hippocampus (Corpuz et al., 2018). It is reported that probiotics can restore neuronal activation indicated by expression of the BDNF, as a key mediator of cognitive behavior in the CA1 region of the hippocampus (Gareau et al., 2011).

### Efficacy of the Microbe Produced Bioactive Transmitters on Brain

The BBB is a highly selective semipermeable border that separates the circulating blood from the brain and extracellular fluid in the CNS, and as a control point, safeguards homeostasis of CNS by firmly controlling the passing molecules and solutes from the bloodstream into the CNS (Castro Dias et al., 2019). The BBB disruption is a crucial component in the pathogenesis of many neurological and neurodegenerative disorders. For a molecule to cross the BBB, it must either be small, lipophilic (non-polar), or a gas. It is because the BBB does not contain the necessary “transport” mechanisms needed to get them across. If not, then the molecule must have a dedicated transporter. However, in some instances, precursor amino acids can cross the BBB.

Despite the gut microbiota or probiotic bacteria produce neurochemicals which can potentially affect activity of neuronal circuits, however, the BBB, as just mentioned, is impermeable or hardly permeable to some of the microbes’ substances. All three catecholamines have dihydroxy benzene ring along with an amine group and are also polar. Therefore, they are unable to diffuse across the membrane, and, hence, they cannot be administered for therapeutic goals (Iversen et al., 2010). Serotonin is also unable to overstep the BBB. It also has long been thought that the BBB would prevent the uptake of GABA (Van Gelder and Elliott, 1958). Therefore, despite accepting their significance, the effectiveness of some neuroactive substances must be explained through mechanisms other than direct effect *via* penetrating the BBB. One possibility is that, *via* nerves and second messengers, signals from the gut can be conveyed directly to the brain (Rhee et al., 2009; Dinan et al., 2013). In addition, regulation of the expression of various genes and the neurotransmitters as well as synaptic-related proteins may be the way by which some gut signals affect the brain (Diaz Heijtz et al., 2011; Distrutti et al., 2014).

### Effect on Expression of Receptors

Glutamate inotropic NMDA receptor is one of the abundant glutamate receptors in the human CNS. A positive relationship is evident between the gut microbiota and the NMDA receptors. Neufeld et al., 2011 showed that expression of the NMDA receptor NR2B subunit is reduced in the hippocampus of the germ-free animals. Similarly, the gut microbiota disruption by antibiotics considerably decreases the NMDA receptor level in the hippocampus (Wang et al., 2015). It has been shown that, in the absence of intestinal bacteria, the central BDNF levels are reduced that, in turn, inhibits the maintenance of NMDA receptor production (Maqsood and Stone, 2016). Treatment with a formulation of inulin and the probiotic bacterium *Enterococcus faecium* increased NMDA/AMPA ratio and induced a robust long-term potentiation, suggesting that the intervention can improve impaired memory (Romo-Araiza et al., 2018).

Few reports also indicate the effect of gut microbiota and probiotics on the GABA receptors. Liang et al. (2017) found that the juvenile gut microbiota disturbances reduced the expression of the α5 and δ subunits of GABA_*A*_ receptors in the hippocampus of adult rats. The study of Boonstra et al. (2015) demonstrated that the treatment of mice by *Lactobacillus rhamnosus* (JB-1) modulates the mRNA expression of GABA_*A*α 2_, GABA_*A*α 1_, and GABA_*B*1*b*_ receptor subunits. Bravo et al. (2011) assessed effect of *Lactobacillus rhamnosus* strain JB-1 on fear conditioning to evaluate the cognitive aspects of anxiety behavior. They found an enhanced memory consolidation along with decreased GABA_*B*1*b*_ mRNA in the hippocampus. They concluded that the probiotic bacterium has a therapeutic potential in modulating the GABA receptor expression (Bravo et al., 2011). It is of noteworthy to point out that reduced excitatory inputs from NMDA receptors onto GABA inhibitory interneurons disinhibit glutamatergic output leading to aberrant synaptic behavior and cognitive deficits (Maqsood and Stone, 2016).

## Intestinal Barrier Integrity

The integrity of intestinal barrier is important for protection against microbial invasion (Ceccarelli et al., 2017). Intestinal mucosa, gut microbiota, immune cells in the mucosa, and various products of epithelial origin are all components of the barrier. Damaged intestinal barrier function may lead to the local or systemic immunological reactions, the degranulation of mast cells, the neuroinflammation, and the activation of vagal nerve afferents (Konig et al., 2016). Prominently, such alterations in the gut environment can damage the protection of the brain against toxic substances. Recent findings indicate that a lot of substances can weaken the integrity of the BBB. This epithelial dysintegrity causes all kinds of molecules such as bacteria, viruses, and protein to enter the brain and threaten its health (Welling et al., 2015). Importantly, this, what is known as “leaky gut,” induces inflammation that can eventually lead to increased permeability of BBB.

It is reported that disturbance of the gut microbiota is directly related to the leaky gut (Jakobsson et al., 2015). For instance, pathogen infection, stress, and antibiotic medication unfavorably impact the gut microbiota leading to increased permeability of the gut. Furthermore, the gut microbiota is also important for the development and the integrity of BBB. It is demonstrated that the BBB permeability is increased in the germ-free mice. The gut microbiota restoration in the germ-free mice decreases the BBB permeability and upregulates the expression of tight junction proteins (Braniste et al., 2014).

Human studies also show that acute stress increases the permeability of intestinal epithelial barrier (Alonso et al., 2012; Vanuytsel et al., 2014) and adversely influence the memory performance (Schoofs et al., 2009). Some other pathways are also conceivable. For instance, it is suggested that increased barrier permeability can induce depressive symptoms (Ait-Belgnaoui et al., 2012), probably by endotoxin-induced inflammatory routes or through direct activation of neural and glial cells that carry Toll-like receptors and respond to a broad variety of microbial products (McCusker and Kelley, 2013).

As the gut microbiota, the probiotics also show protective effects on the intestinal barrier. It is reported that a formulation of probiotics (*Lactobacillus rhamnosus* R0011 and *Lactobacillus helveticus* R0052) attenuated the increased permeability of the colonic barrier in the stressed pup rats (Gareau et al., 2007). *Lactobacillus farciminis* inhibited stress-induced hyperpermeability of the intestinal barrier (Ait-Belgnaoui et al., 2012). In addition, the probiotic bacterium *Lactobacillus plantarum* has been shown to regulate the integrity of intestinal epithelial barrier by stimulation of Toll-like receptor 2 (Karczewski et al., 2010). Given favorable effect of probiotics on improvement of the epithelial barrier function and decreasing its permeability (van Hemert et al., 2013), this mechanism might account for the known beneficial effects of probiotics on the cognitive reactivity.

Moreover, growing evidence indicates that some neurodegenerative diseases with cognitive symptoms are associated with intestinal barrier impairment. It is shown that the intestinal barrier function is impaired in the patients with PD making them susceptible to be exposed to microbial products (Houser and Tansey, 2017). Leblhuber et al. (2015) also reported a leaky intestinal barrier in the patients with AD.

## Short Chain Fatty Acids

Undigested carbohydrates, proteins, and lipids delivered to colon, named “microbiota accessible carbohydrates” undergo microbial degradation and subsequent fermentation (Gareau, 2016). The most important end products of fermentation in the gut are short chain fatty acids (SCFAs) such as butyrate (C4H7O2-), produced especially by *Firmicutes*, propionate (C3H502-) by *Bacteroidetes*, and acetate (C2H402) by anaerobic bacteria. The SCFAs are straight chain saturated fatty acids characterized by a carbon chain length of less than six carbons. Basically, the SCFAs provide the greatest source of energy for intestinal absorptive cells (Topping and Clifton, 2001; Louis and Flint, 2017).

All SCFAs, most importantly butyrate, acts as potent inhibitor of histone deacetylase (Candido et al., 1978; Davie, 2003; Latham et al., 2012). This, in turn, alters the expression of several genes and proteins in the intestinal and neuronal cells (Walsh et al., 2015). Acetate is shown to cross the BBB and reduces its permeability (Deelchand et al., 2009; Frost et al., 2014). Furthermore, the SCFAs contribute to the regulation of numerous immunological and inflammatory responses as well (Abdullahi et al., 2018). While these molecules are not basically known as neuroactive substances, yet, they may affect the neuronal function. The SCFAs display a critical role in protection against oxidative stress, neurotransmission, neurogenesis (Innis, 2007; Maekawa et al., 2009), and enhancing learning and memory (Henriksen et al., 2008; Yurko-Mauro et al., 2010). It is reported that disturbed gut microbiota is associated with reduced concentration of the SCFAs (Feng et al., 2018).

Mechanistically, it is demonstrated that, through inhibiting nuclear factor-kappa beta (NF-κβ) activation, the butyrate suppresses production of the proinflammatory cytokines (Franco-Robles and Lopez, 2015; Aguilar et al., 2016). The butyrate increases the expression of enzymes implicated in the synthesis of the antioxidant glutathione (GSH). By reduction of hydrogen peroxide and lipid hydroperoxide, GSH decreases oxidative stressors that act as neurodegenerative factors (Hamer et al., 2009; Aguilar et al., 2016; Bourassa et al., 2016). At the cellular level, the effects of butyrate are mediated by different receptors, including G-protein-coupled receptors, free fatty acid receptors, and transporters. In addition, the use of butyrate as an energy resource by the beta oxidation pathway could be another action mechanism of the butyrate (Stilling et al., 2015).

The probiotics highly influence production of the SCFAs. It is suggested that the probiotics can improve learning and memory through an increase in the butyrate that, in turn, increases BDNF and decreases the proinflammatory cytokine concentrations in the hippocampus (Romo-Araiza et al., 2018). Treatment with *Clostridium butyricum*, as a butyric acid-producing bacterium, has been shown to improve cognitive deterioration and histopathological changes in the CA1 area of the hippocampus in a mouse model of dementia (Liu et al., 2015). Wall et al. (2012) reported that treating mice with *Bifidobacterium breve* DPC 6330 or *Bifidobacterium breve* NCIMB 702258 had an important effect on the fatty acid composition of the brain. It has been revealed that supplementation with the butyric acid-producing bacteria leads to increased contents of butyrate in the brain (Wall et al., 2012; Liu et al., 2015; Sun et al., 2015) and that improving the memory and learning abilities after consumption of the probiotics might be due to antioxidative effects triggered by the SCFAs (Ho et al., 2019).

It is found that improving learning and memory by butyrate corresponds amplifying the expression of learning associated genes in a mouse model of AD (Govindarajan et al., 2011), and, therefore, it is proposed that the butyrate can be a candidate to treat cognitive impairment (Stilling et al., 2015).

After all, the beneficial effects of SCFAs must be cautiously judged where injurious effects of SCFAs are also reported. Indeed, extreme amount of the SCFAs can cause an inflammatory response and metabolic dysfunction in the body (Ohira et al., 2017). For example, excess concentration of the propionate causes oxidative stress (Fontella et al., 2000), neurotoxicity, and neurological impairments (Khalil et al., 2015). Excessive butyrate is also shown to induce severe cell apoptosis in intestinal epithelium and disruption of the intestinal barrier (Peng et al., 2007).

## Immune Pathways

An important role of the gut mucosa is to mediate innate immunity because it is the first barrier against a large number of specific antigens. Inflammation is principally an adaptive physiological process settled by the immune system in response to pathogens and injury which protects the organism against infection.

### Pro- and Anti-inflammatory Cytokines

One of the biological changes related to inflammation is the activity of cytokines; the proteins that regulate inflammation (Scheller et al., 2011). While the proinflammatory cytokines increase inflammation, the anti-inflammatory cytokines reduce inflammation. However, depending on the condition of secretion, some cytokines such as interleukin-6, may display either anti- or proinflammatory properties (Lyte, 2018). Through modulation of immune system, the gut microbes stimulate the circulating cytokines which, in turn, influence brain function (Banks et al., 1995). Furthermore, it is hypothesized that, beside direct signaling to the brain, the effects of probiotics on memory can be contributed to interaction of the immune system and the enteric immune system (Dantzer et al., 2008). The effect of probiotics on the proinflammatory cytokines has vastly been investigated. Consumption of probiotics, mainly *Bifidobacteria* and *Lactobacilli* strains, reduces the proinflammatory cytokines (Desbonnet et al., 2008, 2010; Rodrigues et al., 2012; Hsiao et al., 2013; Jia et al., 2016; Savignac et al., 2016; Musa et al., 2017) and increases the anti-inflammatory cytokines (Pessi et al., 2000; O’Mahony et al., 2005; Rodrigues et al., 2012).

Excessive proinflammatory cytokines activates the HPA axis and increases the permeability of the BBB, giving rise to some brain dysfunctions (Cowan et al., 2016; Parois et al., 2017). Through augmenting the integrity of the gut barrier, the probiotics reduce inflammation and, hence, prevent bacterial translocation (Zareie et al., 2006; Ait-Belgnaoui et al., 2012). In a study, administration of *Lactobacillus paracasei* PS23 ameliorated memory impairment in the aged SAMP8 mice. This was accompanied by reduced levels of the inflammatory cytokines, TNF-α, and MCP-1, and the generation of the anti-inflammatory cytokine, IL-10, indicating that the intervention preserved brain function by reduction of inflammation level (Huang and Chen, 2018). In addition, the probiotics decrease the proinflammatory cytokines TNF-α, IL-1β, IL-5, IL-6, and IL-8 and increase numbers of natural killer cells, activated lymphocytes, and phagocytosis (Rincon et al., 2014; Wang et al., 2015). It is reported that acquisition of a water maze task and Y-maze alternation were improved in 18-month-old Fischer 344 rats fed with *Lactobacillus pentosus* var. *plantarum* (C29). It was accompanied with the reduction of proinflammatory cytokines, TNF-α and IL-6, indicating that the multispecies probiotic supplement optimized the inflammatory markers (Jeong et al., 2015a).

Whereas normal immunological activity needs a balance between anti- and proinflammatory cytokines, an imbalance of these occur in some neurological disorders (Maier and Watkins, 1998; Dowlati et al., 2010). Bonfili et al. (2017) demonstrated that consumption of a formulation of probiotic (SLAB51) resulted in a significant decrease in plasma level of proinflammatory cytokines of IL-1α, IL-1β, IL-2, IL-12, IFN-γ, and TNF-α, which were higher in AD mice. The probiotic *Lactobacillus farciminis* prevented stress-induced increases in mRNA expression of IL-1β, IL-6, and TNF-α in the hypothalamus (Ait-Belgnaoui et al., 2012). The probiotic bacteria *Lactobacillus helveticus* R0052 and *Lactobacillus rhamnosus* R0011 diminished the IL-1β, IL-8, and TNF-α cytokines (Foster et al., 2011), which are elevated in stress status.

There are other mediators by which the probiotics counteract inflammation. By production of SCFAs like butyrate the probiotics suppress inflammatory cytokines such as IL-1β, TNF-α, and IL-6 (Berni Canani et al., 2012). Furthermore, higher concentrations of BDNF in the symbiotic-treated animals counteract production of the cytokine IL-1β (Carlos and Tong, 2017). Toll-like receptor ligands derived from the probiotics are also shown to display positive effect on production of the anti-inflammatory cytokines (Thomas et al., 2017; Caputi and Giron, 2018). Contrary to these documents, the study of Chen et al. (2019) showed that probiotic administration significantly increased the levels of cytokines IL-1β, IL-2, IL-12, IL-5, IL-6, TNF-α, and IFN-γ.

### Microglia Activity

Microglia, as mononuclear phagocytes, are important for the maintenance of CNS homeostasis. They play a role in the promoting tissue repair, the synaptic pruning, and the recruiting peripheral leukocytes to sites of inflammation in the different areas of brain (Wohleb, 2016; Menard et al., 2017). However, they critically contribute to pathology of CNS where the microglial activation is related to synaptic loss and cognitive dysfunction (Schafer et al., 2012; Stephan et al., 2012; Aguzzi et al., 2013). Neurodegeneration, which is related to mitochondrial defects and oxidative stress, increases proinflammatory cytokines and consequently promotes activity of microglia (Cevenini et al., 2013; Romo-Araiza et al., 2018). Activation of microglia itself leads to the release of proinflammatory mediators and promotes the permeability of the BBB that regulates cerebral homeostasis (Chen et al., 2016; Abdullahi et al., 2018). It is shown that the gut microbiota contributes to regulating the maturation and activation of microglia. The gut microbiota synthesizes considerable amounts of lipopolysaccharides and amyloids that activate the microglia. RNA sequencing of the microglia confirms a substantial difference in the transcriptional profiles between the germ-free and specific pathogen-free mice, which corresponds to the morphology and activity level of the microglia (Pistollato et al., 2016).

## Oxidant/Antioxidant Balance

The major probiotic strains *lactobacilli* and *Bifidobacteria* have a capability to produce potential antioxidants, vitamins, and bioactive molecules (Deguchi et al., 1985; Noda et al., 1994; Pompei et al., 2007; LeBlanc et al., 2013) and, hence, they are able to prevent excessive amounts of free radicals. Thus, they help to attenuate several oxidative stress-associated diseases. Musa et al. (2017) showed that *Lactobacillus fermentum* or *Lactobacillus casei* decreased the oxidants such as NO and MDA and increased the antioxidants such as SOD, GSH, and glutathione peroxidase (GPx). The effect of probiotics on the level of oxidant and antioxidant factors in different animal models of neurological disorders is evaluated. In an animal model of epilepsy, we demonstrated that a probiotic cocktail (*Lactobacillus rhamnosu*s, *Lactobacillus reuteri*, and *Bifidobacterium infantis*) decreased concentration of NO and MDA and increased concentration of total antioxidant capacity (TAC) in the brain (Bagheri et al., 2019). Moreover, the probiotic treatment of the AD rats improved impaired spatial memory, increased the antioxidant index TAC, and reduced the oxidant MDA (Rezaeiasl et al., 2019). In a diabetic animal model, the probiotic administration improved the learning and memory deficit and declined the product of oxidatively damaged DNA, 8-hydroxydeoxyguanosine (8-OHdG) factor, and elevated SOD (Davari et al., 2013). In a clinical trial, a mixture of *Lactobacillus acidophilus*, *Lactobacillus casei*, *Bifidobacterium bifidum*, and *Lactobacillus fermentum* decreased MDA, and the inflammatory protein marker, high-sensitivity C-reactive protein (h-CRP) in AD patients (Akbari et al., 2016). Ho et al. (2019) also showed that probiotic treatment had a favorable effect on balance of the antioxidant enzymes SOD and catalase over the oxidant factor MDA. They concluded that improving the memory and learning abilities after consumption of the probiotics might be due to antioxidative effects triggered by the SCFAs produced by the probiotics (Ho et al., 2019).

## Antiapoptotic Effects

Administration of a probiotic mixture (*Lactobacillus paracasei* ssp. *paracasei* BCRC 12188, *Lactobacillus plantarum* BCRC 12251, and *Streptococcus thermophilus* BCRC 13869) to a D-galactose mouse model of aging improved the learning and memory and reduced activation of the apoptosis mediator caspase-3 in the hippocampus (Ho et al., 2019). Using a mouse model of cerebral hypoperfusion, Rahmati et al. (2019) reported that ingestion of probiotics reduces the hippocampal damage and prevents the spatial learning and memory impairment *via* suppressing apoptosis.

## Pre- and Post-Natal Considerations

Through improving maternal mood, the probiotic intake during pregnancy might influence prenatal and early postnatal bonding to offspring (Alhusen et al., 2012; Goecke et al., 2012). Several studies have reported that the probiotics, with their neuroregulatory and anti-inflammatory properties, may improve the composition and function of gut microbiota in the pregnant mothers (Ait-Belgnaoui et al., 2014; Jiang et al., 2015; Slykerman et al., 2017). Very few researches have paid to find a relationship between the prenatal exposure to probiotic bacteria and the postnatal brain function. It is reported that maternal stress results in altered intestinal microbiota of infants (Zijlmans et al., 2015). In an animal model of stress, we demonstrated that both pre- and postnatal probiotic supplementation improve impaired learning and memory (Hadizadeh et al., 2019).

Altogether, the action mechanisms of probiotics seem to be very complex and under a wide range of molecules and systems, strengthening what is known as microbiota-gut-brain axis. Furthermore, very importantly, sophisticated interactions between different mechanisms discussed here sound more in complexity of the axis function than might be imagined. Therefore, future investigation must search action mechanisms of single bacterial strains to comprehend their specific potential on the cognitive functions; however, understanding cross-talk between the different mechanisms is also appreciated.

## Future Perspectives

By revelation of these connections, research on the gut and probiotic bacteria hopefully offers new explanations of the mental health and potential avenues of treatment (Hooks et al., 2018). Close relationship between several brain diseases and the gut microbiota alterations offer the intriguing assumption that the gut microbiota can be used as biomarker to assist in the diagnosis of the brain disorders. Is it possible to replace aged and/or damaged microbiota with appropriate probiotic bacteria to prevent or treat the diseases (Shen et al., 2018)? Although current evidences are promising, however, at least regarding the cognitive function, we are just beginning. Meanwhile, some discrepancies exist between preclinical and clinical findings which impede clinical application of probiotics. Hence, more research is required to further explore the potential effects of probiotics on the cognitive functions in clinical populations. Another concern is that, in human researches, evidence to show that probiotics are helpful in people with normal intestinal microbes is scant. Therefore, regarding the use of the probiotics, whether as supplementation for health or as therapeutic tool, several considerations are necessary to be addressed. The biological effects of the probiotic bacteria are strain specific (Stenman et al., 2020), and, therefore, success or failure of one strain cannot be attributed to another one. Thus, a case-by-case approach must be considered to examine the beneficial actions of specific strains of probiotics. Furthermore, preclinical studies appear that gender- and person-specific traits can also define the success of probiotic supplementations (Kiousi et al., 2019). In this regard, using novel molecular and based technologies for proper strain identification is imperative (Azais-Braesco et al., 2010). Indeed, majority of researchers have administered multistrain probiotic formula, however, evidence shows that even the strains related to the same species of bacteria differentially affect their host (McFarland et al., 2018). This approves that the beneficial bacteria display highly strain-specific effects (Savignac et al., 2015), and to gain a maximum efficacy of the probiotics, future researches must focus on optimal administration routes as well as strain, host, and sex specificity of bacteria (Athari Nik Azm et al., 2018). Other considerations such as the ages with the most susceptibility to probiotics and the duration of intervention are also necessary to be addressed (Gonzalez-Arancibia et al., 2019). Yet, how convenient to administer probiotics, cost public acceptance and specially risk of side effects required further investigations. On the other hand, the use of different probiotics for patients with immunological problems or leaky gut has been accompanied with infections, sepsis, fungemia, and bacteremia. Therefore, it is necessary to be cautious in selecting probiotics for clinical practice and a risk-benefit ratio must be considered (Fijan, 2014). Altogether, the area of bacteriotherapy opens a new therapeutic window in which neuroactive-producing probiotics play a key role in the treatment of cognitive dysfunction neurodegenerative and neuropsychiatric disorders.

## Author Contributions

The author confirms being the sole contributor of this work and has approved it for publication.

## Conflict of Interest

The author declares that the research was conducted in the absence of any commercial or financial relationships that could be construed as a potential conflict of interest.
